# Rate and Determinants of Recurrence at 1 Year and 5 Years After Stroke in a Low-Income Population in Rural China

**DOI:** 10.3389/fneur.2020.00002

**Published:** 2020-01-23

**Authors:** Jing Han, Wenjing Mao, Jingxian Ni, Yanan Wu, Jie Liu, Lingling Bai, Min Shi, Jun Tu, Xianjia Ning, Jinghua Wang

**Affiliations:** ^1^Department of Neurology, Tianjin Medical University General Hospital, Tianjin, China; ^2^Department of Neurology, The Affiliated Hospital of North China University of Science and Technology, Tangshan, China; ^3^Laboratory of Epidemiology, Tianjin Neurological Institute, Tianjin, China; ^4^Tianjin Neurological Institute, Key Laboratory of Post-Neuroinjury Neuro-repair and Regeneration in Central Nervous System, Ministry of Education and Tianjin City, Tianjin, China; ^5^Department of Neurology, Liaocheng People's Hospital, Liaocheng, China

**Keywords:** stroke, recurrence, predictors, epidemiology, incidence

## Abstract

Recurrent stroke is becoming an increasingly important public health issue owing to the increased risk of disability and death. However, population-based studies investigating the rate of recurrent stroke in China are rare. We explored the rate and determinants of recurrent stroke within 1 and 5 years after the initial stroke in a rural population in China. Data for stroke events were obtained from the Tianjin Brain Study, conducted between 1992 and 2016. The age-standardized rates of recurrent stroke within the first year and the first 5 years after the initial stroke were calculated for this period. Determinants of recurrent stroke were assessed using Cox regression analyses. The overall age-standardized rate of recurrent stroke within 1 year was 5.7% (men, 6.9%; women, 4.6%); within 5 years, the overall recurrent stroke rate was 22.5% (men, 24.0%; women, 20.2%). The recurrence rate increased with advancing age and decreased with increased educational attainment. Age ≥65 years and a history of alcohol consumption were independent risk factors for recurrent stroke within 1 year after the incident stroke, after adjusting for age, sex, education, hypertension, diabetes, smoking, and alcohol consumption. However, the risk of recurrent stroke within 5 years after the incident stroke was positively associated with male sex, age ≥65 years, a lower level of education, known diabetes, and alcohol consumption, after adjusting for the previously indicated covariates. These findings suggest a crucial need to address risk factor management among stroke patients to reduce the burden of stroke, especially among low-income populations. Furthermore, a multicenter, large sample, nationwide study is urgently needed.

## Introduction

Stroke is one of the leading causes of death and disability, worldwide (1) and is responsible for ~4.4 million deaths (9% of total) annually (2). Over the past two decades, there has been a notable increase (84%) in the absolute numbers of stroke survivors globally although the incidence of stroke has decreased in high-income countries (3). However, the number of stroke survivors under 75 years old was almost 30% higher in low- and middle-income countries than in high-income countries (3). The risk of recurrent stroke is becoming increasingly important owing to its associated high risk of disability and death (4–9), and the risk varies according to the elapsed time since the incident stroke (10–15). The population-based cumulative risk of recurrent stroke during the 5 year period after the incident stroke has been reported to be 19% in Manhattan (USA), 29% in Rochester (USA), 30% in Oxfordshire (UK), and 32% in Perth (Australia) (16–19).

Each year, there are ~2.5 million new stroke cases reported in China, and currently there are ~7.5 million stroke survivors; nationally, the stroke recurrence rate remains high (11.2%) (20). Over the past few decades, the incidence of stroke has increased in China both in urban and rural populations (21–25). Thus, recurrent stroke prevention is of considerable importance to both individuals and overall public health. However, few population-based studies have described the incidence of recurrent stroke in China, especially among rural populations. Therefore, in this study, we aimed to assess the rate of recurrent stroke within 1 and 5 years after the incident stroke in rural China.

## Methods

### Study Population

This study was a population-based stroke surveillance study that began in 1985 in Tianjin, China. The study design has been previously described (23–25). Briefly, it included 15,438 residents living in a township in Tianjin, China, where 95% of the adults were low-income farmers. The primary source of income was grain production, and the annual per capita income was <$100 US in 1990 and <$2000 USD in 2015 (26). In 1991, the illiteracy rates among residents 35–74 years old were 30% for men and 40% for women. The population characteristics remained stable over the study period (27). Since neuroimaging technology became available in 1992, this study analyzed recurrent stroke events since that time.

The study protocol was approved by the ethics committee of Tianjin Medical University General Hospital (TMUGH); written informed consent was obtained from each participant.

### Stroke Definition and Types

All included stroke events were symptomatic and diagnosed using pre-established criteria, including clinical features and imaging evidence (MRI or CT scan). The initial (incident) stroke was defined as the first occurrence of rapidly developing signs of focal neurologic disturbances of presumed (no other apparent cause) vascular etiology lasting >24 h (28). Transient ischemic attacks (TIAs) and silent strokes (diagnosed by imaging only) were excluded, and stroke cases with TIA histories were regarded as incident events. Patients with transient symptoms and concurrent neuroimaging evidence of brain infarctions were considered to be stroke cases, based on the “tissue” definition (29). Recurrent stroke was defined as a new stroke event occurring at least 28 days after the incident event.

The stroke types included ischemic stroke (IS), intracerebral hemorrhage (ICH), and unknown. IS was defined as a thrombotic brain infarction, cardioembolic stroke, or lacunar infarct.

### Event Ascertainment Processes

All incident stroke events included first-ever and recurrent strokes registered according to the previously published procedure (25). Stroke event determinations were performed using a predetermined procedure. First, local village physicians reported stroke events (both initial and recurrent) to the community hospital within 24 h of onset. Second, community hospital physicians then visited the surviving patients' homes to confirm stroke events and obtain clinical feature information within 72 h. Confirmed stroke events, diagnosed using imaging modalities, were reported each month to neurologists at TMUGH. Suspected events (no imaging performed) were reported in a timely manner. Finally, the TMUGH neurologist identified possible stroke events via door-to-door interviews as soon as possible. To ensure that all incident stroke events were registered, three sources were used to obtain information regarding stroke events. First, local physicians reported events according to a predefined procedure. This was the main information resource, as the local physicians were the first medical professionals to contact the patients. Second, in cases involving inpatient stroke events, the medical records were obtained from the hospital or insurance company. Third, information from the all-cause death registry supplemented stroke events missing from the registry.

Information regarding stroke onset was obtained during interviews conducted by the community hospital physicians and the TMUGH neurologist; information included demographics, time of stroke onset, clinical signs, and previous stroke status. Other information, including imaging characteristics, prescribed therapy, and post-discharge outcomes, was obtained by the TMUGH neurologist during interviews with survivors, their family members, or a local health worker.

The recruitment period was from January 1, 1992, to December 31, 2016, and follow-up was completed on December 31, 2017. During the surveillance period, all stroke events and all-cause deaths were registered and followed. The dates of death or emigration from the township were determined using population registries. All changes in demographic information were recorded, including births, deaths, immigrations (including that due to marriages), and emigrations (including that due to entering a high school or university or working in the city). Peasant workers were included in this study because all residents working in the city were seasonal workers who regularly returned to the township during traditional festivals and the farming season.

### Inclusion and Exclusion Criteria

Only patients who survived the index event were entered into the present analysis. Death was considered to have been caused by the incident stroke if it occurred within 28 days of symptom onset. All patients with at least 1 year of follow-up after their first-ever stroke events were included in this study. Follow-up evaluations were conducted every month, and recurrent strokes were classified using information from interviews, conducted directly during follow-up visits or by telephone calls to patients, next of kin, witnesses, or the attending physicians.

All patients with TIAs; suspected stroke deaths without imaging evidence or confirmation by a TMUGH neurologist; and silent strokes, detected only by imaging, were excluded from this study. Patients suffering neuroimaging-confirmed strokes underwent computed tomography or magnetic resonance imaging examinations in the county central hospital.

### Statistical Methods

Categorical variables are presented as numbers (%), and continuous variables are presented as means (standard deviations, SD). Patient ages were categorized into 3 subgroups (<45, 45–64, and ≥65 years) when the demographic features of this population were described and the rates of recurrent stroke were assessed; however, only 2 subgroups (<65 and ≥65 years) were used to assess the determinants of recurrent stroke. Education attainment was categorized into 3 subgroups (0, 1–6, and ≥7 years). Smoking status was categorized into 3 subgroups (never smoker, past smoker, and current smoker); similarly, drinking status was also categorized into 3 subgroups (never drinkers, past drinkers, and current drinkers). The recurrent stroke incidence was calculated as the cumulative frequency of recurrent events at 1 and 5 years after the incident stroke. Association of education attainment with the risk of the recurrent stroke was assessed by Kaplan-Meier survival analysis and presented as log rank.

Multivariable Cox regression analyses were used to identify the predictors of recurrent stroke within both time periods after adjusted by covariates (sex, age, education attainment, stroke types, previous history of hypertension and diabetes, smoking and drinking status); the risk of recurrent stroke occurrence is presented as adjusted hazard ratios (HRs) and 95% confidence intervals (CIs). The follow-up time was recorded in years and was calculated as the interval between the date of the initial stroke and the date of the recurrent stroke for those patients experiencing recurrent strokes within 1 and 5 years after the initial stroke. For those patients without recurrent strokes within 1 or 5 years after the initial stroke, the follow-up time was defined as 1 or 5 years, respectively. Moreover, for patients who died within 28 days after the initial stroke, the follow-up time was defined as the interval between the initial stroke and the date of death. During the study period, stroke patients who experienced initial strokes before immigrating to the township were included in the study; stroke patients who emigrated from the township after the initial stroke were excluded from the study. However, in this study, no stroke patients immigrated to or emigrated from the township during the study period. SPSS version 15.0 for Windows (SPSS, Chicago, IL, USA) was used for the analyses; statistical significance was defined as *P* < 0.05.

## Results

### Patient Characteristics

A total of 1,185 individuals experienced initial stroke events, including ICH, IS, and undefined events between 1992 and 2016. All 1,185 patients were included in the determination of the rate and associated predictors of recurrent stroke 1 year after the incident event; patients experiencing incident strokes between January 1, 1992, and December 31, 2012 (*n* = 899), were included in the determination of the 5 year recurrent stroke rate and its associated predictors.

Between 1992 and 2016, 1,185 initial stroke events were included in this study; the mean time to the first recurrent stroke within the first year was 0.82 years; for the 5 year time frame, the mean time to recurrence was 3.08 years. In both study periods, patients experiencing initial strokes were more likely to be men, older, and less educated. Further, IS was the most common stroke event during these periods (Table 1).

**Table 1:** Descriptive characteristics of patients with first-ever stroke by time period in this study.

**Groups**	**1992–2016**	**1992–2012**
**SEX**, ***N*** **(%):**		
Men	707 (59.7)	537 (59.7)
Women	478 (40.3)	362 (40.3)
Total	1,185 (100)	899 (100)
**AGE AT STROKE ONSET, MEAN (SD), YEARS**		
	65.49 (11.67)	65.54 (11.94)
**AGE GROUPS**, ***N*** **(%)**		
<45 years	54 (4.6)	82 (9.1)
45–64 years	506 (42.7)	422 (46.9)
≥65 years	625 (52.7)	395 (43.9)
**EDUCATION ATTAINMENT, MEAN (SD), YEARS**		
	3.35 (3.28)	2.89 (3.19)
**EDUCATION GROUPS**, ***N*** **(%):**		
0 years	437 (36.9)	390 (43.4)
1–6 years	570 (48.1)	407 (45.3)
≥7 years	178 (15.0)	102 (11.3)
**SUBTYPES OF FIRST-EVER STROKE**, ***N*** **(%)**		
Ischemic stroke	916 (77.3)	671 (74.6)
ICH	231 (19.5)	192 (21.4)
UND	38 (3.2)	36 (4.0)
**DIAGNOSIS BY IMAGING FOR FIRST-EVER STROKE**, ***N*** **(%):**		
Ischemic stroke	726 (79.3)	498 (74.2)
ICH	218 (94.4)	178 (92.7)
Total	844 (71.2)	676 (75.2)
**RECURRENT STROKE EVENTS**, ***N*** **(%)**		
IS	45 (63.4)	112 (55.4)
ICH	12 (16.9)	31 (15.3)
Unknown	14 (19.7)	59 (29.2)
Total	71 (100)	202 (100)
**DIAGNOSIS BY IMAGING FOR RECURRENT STROKE, N (%):**		
Ischemic stroke	38 (84.4)	97 (86.6)
ICH	11 (91.7)	22 (71.0)
Unknown	0 (0)	1 (1.7)
Total	49 (69.0)	120 (59.4)

*SD indicated standard deviation; 95% CI indicates 95% confidence interval*.

*ICH, Intracerebral hemorrhage; IS, Ischemic stroke; UND, Undefined stroke*.

### Recurrent Stroke Rate

During the study period, 71 recurrent strokes occurred in the 1 year follow-up group and 202 occurred in the 5 year follow-up group. The overall rate of recurrent stroke within 1 year was 5.7% (men, 6.9%; women, 4.6%); in the 5 year group, the overall rate of recurrent stroke was 22.5% (men, 24.0%; women, 20.2%).

Table 2 shows an increasing rate of recurrent stroke that corresponds with increasing patient age at the time of the initial stroke onset; compared with patients aged <65 years old, the rate of recurrent stroke among patients ≥65 years old was higher (7.4%, *P* = 0.037) in the 1 year follow-up group.

**Table 2:** Age-adjusted rates of recurrent stroke by subtypes [% (95% CI)].

**Groups**	**Recurrence rate within 1 year**	**Recurrence rate within 5 years**
**SEX:**		
Men	6.9 (5.1, 8.8)	24.0 (20.4, 27.6)
Women	4.6 (2.7, 6.5)	20.2 (16.0, 24.3)
Total	5.7 (4.4, 6.9)	22.5 (19.7, 25.2)
**SUBTYPES OF FIRST-EVER STROKE:**		
IS	6.2 (4.7, 7.8)	24.4 (21.2, 27.7)
ICH	6.1 (3.0, 9.2)	19.8 (14.1, 25.5)
Other	0	0
**AGE GROUPS:**		
<45 years	0	10.6 (1.5, 19.8)
45–64 years	4.9 (3.0, 6.8)	23.8 (19.4, 28.2)
≥65 years	7.4 (5.3, 9.4)	22.6 (18.9, 26.4)
**EDUCATION: ATTAINMENT:**		
0 year	5.3 (3.2, 7.4)	18.2 (14.4, 22.1)
1–6 years	6.8 (4.8, 8.9)	28.5 (24.1, 32.9)
≥7 years	5.1 (1.8, 8.3)	14.7 (7.7, 21.7)
**PREVIOUS DISEASE: HISTORIES:**		
**Hypertension**		
Yes	6.2 (4.8, 7.7)	22.9 (20.0, 25.8)
No	4.2 (0.9, 7.5)	19.2 (11.3, 27.1)
**Diabetes**		
Yes	9.4 (4.5, 14.3)	39.0 (28.2, 49.8)
No	5.5 (4.2, 6.9)	20.8 (18.0, 23.6)
**LIFESTYLE:**		
**Smoking status**		
Never smoked	5.7 (3.9, 7.6)	22.3 (18.5, 26.2)
Ever smoked	6.5 (1.4, 11.7)	30.0 (19.0, 41.0)
Current smoking	6.3 (4.1, 8.4)	21.2 (17.1, 25.4)
**Drinking status**		
Never drank	5.7 (4.1, 7.2)	22.1 (18.9, 25.4)
Ever drank	11.5 (4.7, 18.3)	43.9 (30.6, 57.1)
Current drinker	5.3 (2.7, 7.9)	17.9 (12.8, 23.0)

**Figure 1:**
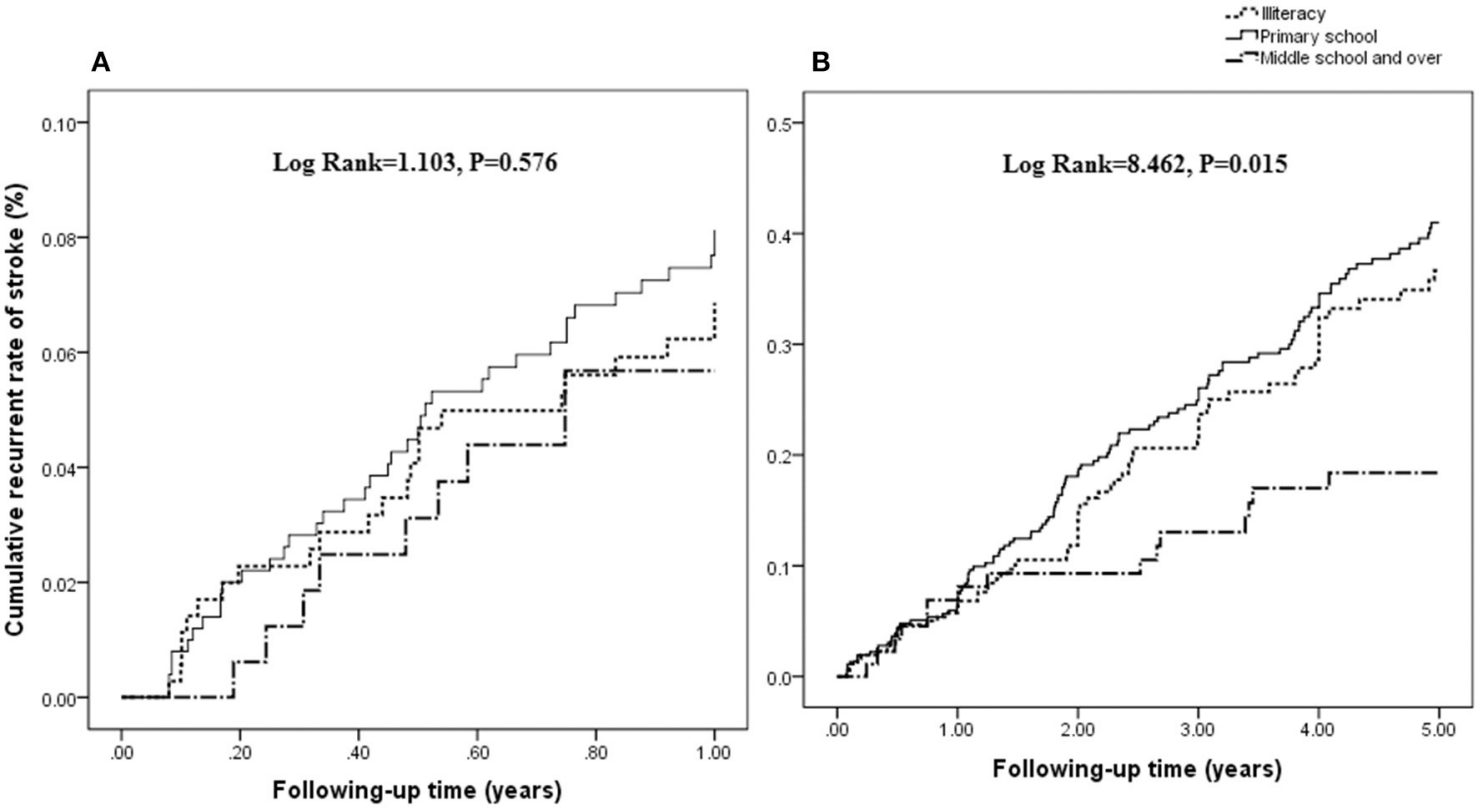
Association between the rate of recurrent stroke and education attainment. There was no significant association between the rate of recurrent stroke within 1 year and education attainment (log rank = 1.103; *P* = 0.576; **A**). However, a negative association is shown between the rate of recurrent stroke within 5 years and education attainment (log rank = 8.462; *P* = 0.015; **B**).

There was no significant association between the rate of recurrent stroke within 1 year and education attainment (log rank = 1.103; *P* = 0.576; Figure 1A). However, there was a negative association between the rate of recurrent stroke within 5 years and education attainment (log rank = 8.462; *P* = 0.015; Figure 1B).

### Determinants of Stroke Recurrence

Compared with patients aged <65 years old, the risk of stroke recurrence within 1 year after the initial stroke more than doubled among patients ≥65 years (*P* = 0.004) after adjusting for age, sex, education level, hypertension, diabetes, smoking status, and drinking status; there was a lower risk of recurrence for those experiencing an IS than for those with an initial ICH. The risk of recurrent stroke within 5 years after the incident stroke was 1.65-fold higher among men than among women (*P* = 0.017). Compared with patients aged <65 years, the risk of recurrence in patients ≥65 years old increased 1.54-fold (*P* = 0.009). Similarly, the risk of recurrence in patients with <7 years of education was almost double that of patients with at least 7 years of education (*P* = 0.017). The risk was also 1.71-fold higher among those with a previous diabetes diagnosis than among those without diabetes (*P* = 0.008), and the risk of recurrence was more than two-fold higher among patients who ever drank compared to those who never drank (*P* = 0.008; Table 3).

**Table 3:** Determinants of recurrent stroke at 1 and 5 years after the first-ever stroke among the study population (HR with 95% CI).

**Predictors**	**Reference**	**Overall**		**<65 years**		**≥65 years**	
		**Within 1 year**	**Within 5 years**	**Within 1 year**	**Within 5 years**	**Within 1 year**	**Within 5 years**
Sex:	Women						
Men		1.67 (0.85, 3.26)	1.65 (1.09, 2.50)^*^	1.75 (0.47, 6.44)	1.79 (0.82, 3.89)	1.59 (0.72, 3.47)	1.55 (0.94, 2.55)
Age groups:	<65 years						
≥65 years		2.29 (1.31, 4.00)^*^	1.54 (1.11, 2.12)^*^	—	—	—	—
Education attainment:	≥7 years						
0 year		0.88 (0.37, 2.11)	1.55 (0.83, 2.87)	0.36 (0.04, 3.03)	0.91 (0.37, 2.23)	2.29 (0.30, 17.37)	—
1–6 years		1.21 (0.57, 2.58)	1.96 (1.13, 3.42)^*^	1.10 (0.46, 2.62)	1.90 (1.07, 3.38)^*^	2.96 (0.40, 22.16)	—
First-ever stroke types:	ICH						
IS		0.53 (0.29, 0.96)^*^	0.70 (0.48, 1.01)	0.63 (0.24, 1.64)	0.63 (0.39, 1.03)	0.43 (0.20, 0.94)^*^	0.73 (0.41, 1.29)
Hypertension:	No						
Yes		1.39 (0.59, 3.26)	0.98 (0.60, 1.60)	1.16 (0.27, 5.01)	0.89 (0.38, 2.09)	1.65 (0.57, 4.74)	1.17 (0.64,2.12)
Diabetes:	No						
Yes		1.73 (0.93, 3.22)	1.71 (1.15, 2.54)^*^	3.19 (1.30, 7.79)^*^	2.95 (1.75, 4.96)^*^	1.11 (0.43, 2.88)	0.88 (0.45, 1.73)
Smoking status:	Never						
Past smoker		0.36 (0.11, 1.15)	0.52 (0.27, 1.00)	0.76 (0.11, 5.35)	0.63 (0.23, 1.78)	0.21 (0.05, 0.98)^*^	0.43 (0.16, 1.14)
Current smoker		0.84 (0.43, 1.65)	0.70 (0.46, 1.06)	0.59 (0.13, 2.70)	0.73 (0.32, 1.63)	0.95 (0.45, 2.01)	0.70 (0.42, 1.15)
Drinking status:	Never						
Past drinkers		2.46 (0.99, 6.11)	2.23 (1.24, 4.01)^*^	1.82 (0.35, 9.42)	1.74 (0.77, 3.92)	3.60 (1.18, 11.01)^*^	3.09 (1.19, 8.05)^*^
Current drinkers		0.90 (0.44, 1.84)	0.86 (0.56, 1.32)	1.41 (0.36, 5.53)	0.92 (0.48, 1.79)	0.70 (0.28, 1.75)	0.73 (0.39, 1.37)

* *P < 0.05 compared to reference*.

### Determinants of Stroke Recurrence Stratified by Age

Table 3 shows the determinants of recurrent stroke after stratifying the population by age. A history of diabetes was the most common risk factor for recurrent stroke within 1 and 5 years after the initial stroke among patients aged <65 years; the recurrence risk increased 2-fold compared to patients without diabetes. Moreover, the risk of recurrence within 5 years was 1.9-fold higher among those with 1–6 years of education compared to those with more than 6 years of education (*P* = 0.028).

Among patients aged ≥65 years, the risk of recurrence within the first post-stroke year was 57% lower in patients experiencing initial IS than in those experiencing initial ICH (*P* = 0.033); this patient group also had a 79% lower risk of recurrent stroke among those who ever smoked than among those who had never smoked (*P* = 0.046). However, there was a 3.6-fold higher risk of recurrence for those who ever drank than among those who never drank (*P* = 0.025). The risk of recurrence within 5 years increased >2-fold among patients who ever drank compared to that among those who never drank (*P* = 0.021).

## Discussion

This is the first population-based report evaluating the recurrent stroke rate in rural China. In this study, we assessed the rate of recurrent stroke within 1 (5.7%) and 5 (22.5%) years after the first-ever stroke. The recurrence rate over both time periods increased significantly with advancing age. After adjusting for age, sex, education level, hypertension, diabetes, smoking, and alcohol consumption, advanced age was an independent predictor of recurrent stroke within both time frames among the overall population. Moreover, male patients, patients with lower educational attainment, and those with a known diabetes history had higher risks of recurrent stroke within the 5 year period. Further, age-stratified analysis showed that known diabetes history was an independent risk factor of stroke recurrence at both 1 and 5 years after the initial stroke among stroke patients aged <65 years old. Moreover, patients with a lower educational attainment (1–6 years) had a high risk of stroke recurrence. However, for patients aged 65 years and over, ever drinking was an independent risk factor of recurrent stroke at both 1 and 5 years after initial stroke onset. In addition, experiencing initial IS and ever smoking were independent protective factors for risk of stroke recurrence among elderly individuals.

The stroke recurrence rate remains controversial. The risk of stroke recurrence is reported to range from 7.0 to 20.6% over the first year (4, 12, 13), from 16.2 to 35.3% over the first 5 years (4, 10, 14), and from 14 to 51.3% over the first 10 years after the initial stroke (4, 10, 15). A population-based cohort study from the Middle East indicated that the cumulative incidence of stroke recurrence was 14.5% by the end of 5 years, with the highest rate occurring during the 1st year after the initial stroke (5.6%) (29). In the present study, the 1 and 5 year recurrence rates were 5.7 and 22.5%, which are within the range reported previously. This finding suggests that more than 20% of patients will suffer a recurrent stroke within the first 5 years. The relatively low recurrence rates observed in this study are most likely due to the relatively young (average age <65 years) study population.

The disparity among the reported recurrence rates may be partially explained by the differing definitions of stroke recurrence. There is wide variation in the definition of recurrent stroke, ranging from any focal neurological deficit lasting >24 h and occurring after an initial stroke (14, 16, 18, 30) to an event occurring >28 days after an initial stroke (31, 32). In the present study, recurrent stroke was defined as a stroke occurring >28 days after the incident stroke.

In the present study, age was identified as a predictor of recurrent stroke. A previous study from northern Sweden indicated that the risk of stroke recurrence rose by 3% for every additional year of age (8). A similar trend was reported in a study from the Middle East where a 2% increase in risk was observed for every additional year of age (29). Another study suggested that the risk of recurrence significantly increased among patients >75 years old compared to patients <65 years old; the risk of recurrence increased by 13% in patients 76–85 years old and by 16% in those >85 years old (33). In the present study, relative to patients who were <65 years old, we observed a 129% and a 54% increase in the 1 and 5 year risks, respectively, of recurrent stroke among patients ≥65 years old.

Reports of sex-based differences in stroke outcomes are also inconsistent. Several previous studies have reported worse outcomes among women than among men, with women demonstrating greater functional impairment, mortality, recurrence, and dependency rates 3 and 12 months post-stroke (1, 34, 35). However, other studies have not reported these sex-based differences (36). In the present study, there was a higher risk of recurrence among men than among women within the 5 year period after the incident stroke, but this difference was not evident within the first year. In this population, the young age of stroke onset and the poor management of post-stroke risk factors among men may have contributed to the observed sex-based differences in recurrence.

There is strong evidence for an inverse relationship between socioeconomic status (SES) and stroke incidence and mortality (37–39). However, evidence for a potential association between SES and stroke recurrence is limited (39). Previous studies suggested that this relationship was sex-based (40, 41), but no significant association between SES and stroke recurrence was found in another population-based-study (10). In the present study, a higher risk of recurrent stroke within 5 years after the incident stroke was observed in patients with lower levels of education (1–6 years) than in those with higher levels of education (≥7 years) in the overall population and in those aged <65 years old. However, the risk of recurrent stroke within 5 years after the incident stroke was not higher in those without education attainment (illiteracy) in the overall population and among those aged <65 years old; the lower number of patients with higher levels of education (≥7 years) in the overall population (11.3%) and the lower number of patients with lower levels of education among those aged <65 years old (15%) may partly explain this disparity. Furthermore, the higher percentage of female patients in the illiterate group may contribute to the association of lower education levels with poor stroke outcomes. Poor risk factor management and limited access to medicine among those with lower educational attainment and income may partially explain this relationship (42, 43).

The association between smoking and stroke risk remains controversial. In the Framingham Heart Study, smoking cessation was associated with a significantly lower risk of cardiovascular disease within 5 years among heavy smokers relative to current smokers; however, relative to never smokers, former smokers' cardiovascular disease risk remained significantly elevated beyond 5 years after smoking cessation (44). Another study from the Intracerebral Hemorrhage Outcomes Project found that the functional outcomes among patients with intracerebral hemorrhage were similar between recent smokers and former smokers (45). In the present study, a lower risk of recurrent stroke was found in patients who had ever smoked in the overall population and among elderly stroke patients within 1 year after their initial stroke. The neuroprotective effect of nicotine might explain this finding partly (46–48). The exact cause however needs further research.

Heavy alcohol consumption is well-known risk factor for stroke (49, 50), but the relationship between the risk of recurrent stroke and alcohol consumption remains unknown, especially in China. Alcohol consumption independently predicted impairment 2 years after stroke in one study (51), but no association with functional outcomes after stroke was found in other studies (52–54). In this study, ever drinking was an independent risk factor for recurrence in the overall population and among elderly stroke patients at both 1 and 5 years after the initial stroke. In this population, patients drank in the past ceased drinking alcohol after developing severe diseases; this may explain the positive association between ever drinking and stroke recurrence.

Several studies have suggested that diabetes mellitus is an important predictor of recurrent stroke (55–57). Consistent with these studies, in the present study, we observed a significant association between diabetes mellitus and stroke recurrence for the whole study population and those patients aged <65 years.

This study has several limitations. First, the study population was from a township in northern China, which is not representative of the overall population of China. However, the prospective study design and long study period may have decreased recall and selection bias. Moreover, the 362,596 person-years of total follow-up fulfill the 100,000 person-years of follow-up criterion for population studies (58). Second, we did not collect detailed information regarding dietary habits and medication use; therefore, other possible determinants of stroke recurrence could not be assessed in this study. Third, data on smoking and alcohol consumption were not available in this study; this may affect the specific evaluation of the relationship between smoking or alcohol consumption and stroke recurrence. Fourth, our study lacks data on medication usage. However, in this low-income population, the rate of using medicine was lower; thus, the impact of medication usage on stroke recurrence may be ignored. Finally, information regarding medical care among these stroke patients was not available. This was a low-income, poorly educated population, and few had medical insurance before 2008. Thus, they would have been taken care of by family members, including spouses, children, siblings, and others. Only unmarried men who suffered strokes were sent to the official nursing home.

## Conclusions

This report assessed the rate of recurrent stroke in rural China using a population-based study design. The rates of recurrent stroke within 1 and 5 years after the first-ever stroke were 5.7 and 22.5% and increased significantly with advancing age. The determinants of stroke recurrence were associated with age. After adjusting for covariates, a known diabetes history was an independent risk factor for stroke recurrence at both 1 and 5 years after the initial stroke among patients aged <65 years old; moreover, lower educational attainment (1–6 years) increased the risk of stroke recurrence. However, for patients aged 65 years and over, ever drinking was an independent risk factor for recurrent stroke at both 1 and 5 years after the initial stroke onset; experiencing an initial IS and ever smoking were protective factors for the risk of stroke recurrence. These findings suggest a crucial need to address risk factor management among stroke patients to reduce the burden of stroke, especially among low-income populations. Furthermore, a multicenter, large sample, nationwide study is urgently needed.

## Data Availability Statement

All datasets generated for this study are included in the artical/supplementary material.

## Ethics Statement

The study protocol was approved by the ethics committee of Tianjin Medical University General Hospital (TMUGH); written informed consent was obtained from each participant.

## Author Contributions

JW and XN contributed to the study design, performed data collection, data interpretation, and critical review. JW performed data analysis. JH and WM contributed to drafting of the article. JH, WM, JN, YW, JL, LB, MS, and JT performed data collection, case diagnoses, and confirmation of case diagnoses. All authors read, revised, and approved the final version of the paper.

### Conflict of Interest

The authors declare that the research was conducted in the absence of any commercial or financial relationships that could be construed as a potential conflict of interest.
